# A national survey data for the technical and economic assessment of African catfish production in Nigeria before and during the COVID-19 period

**DOI:** 10.1016/j.dib.2023.109917

**Published:** 2023-12-06

**Authors:** Olanrewaju Femi Olagunju, Dadi Kristofersson, Tumi Tómasson, Theódór Kristjánsson

**Affiliations:** a Federal Department of Fisheries and Aquaculture, Abuja, Nigeria; b Department of Economics, School of Social Sciences, University of Iceland, Reykjavík, Iceland; c UNESCO GRÓ-Fisheries Training Programme, Marine and Freshwater Research Institute, Hafnarfjörður, Iceland; d Marine and Freshwater Research Institute, Hafnarfjörður, Iceland

**Keywords:** Aquaculture, Production management, Production scale, Experience, Markets, Feed types, Profitability, Production constraints

## Abstract

This data article presents a dataset obtained from a national survey of African catfish production in Nigeria. The African catfish is an important aquaculture species in various regions in the world and it is, after Tilapia, the most commonly cultured fish in Africa. Nigeria's share in the global production of African catfish exceeds 67 %. The dataset encompasses data collected from ten major catfish-producing states in Nigeria, with a focus on two distinct periods: before and during the COVID-19 pandemic. A total of 609 operations were captured for the pre-COVID and 509 for the COVID period. The dataset includes a wide array of variables, covering the cost and quantities of inputs and outputs, socioeconomic factors, market dynamics, feed types, challenges faced by farmers, scale of production, and farmers' level of experience. It offers valuable insights and opportunities for various stakeholders. Researchers can utilize it to explore production performance, resilience, and adaptation strategies. Industry players, including catfish farmers and suppliers, can make data-driven decisions to enhance their operations. Policymakers can formulate evidence-based policies to support sustainable growth in the catfish farming sector. Other developing countries can draw lessons from Nigeria's experiences to bolster their aquaculture sectors.

Specifications Table

**Table:** 

Subject	Agricultural Sciences/ Aquaculture
Specific subject area	Economic assessment, production management and profitability dynamics
Data format	Cleaned raw data, partly analysed data, questionnaire template.
Type of data	xlsx file (data set with numbers and strings).xlsx file (questionnaire template for the Open Data Kit - ODK).pdf file (PDF format of the survey form)
Data collection	The survey was carried out from May 2021 to February 2022 through interviews by pre-trained enumerators using an electronic questionnaire – open-data-kit (ODK). A multi-stage sampling technique was used. The 10 states were purposively selected while the farmers within each state were randomly selected from lists provided in the states. The elicited information consisted of the production cost and quantity, socioeconomic information, pond types, major feed used, market, constraints, sources of technical support variety of and other variables. The production data collected is based on a cycle and therefore the fixed cost was prorated to a cycle and the production scale to a year (details in the data description section).
Data source location	Country: Nigeria City/Town/Region: Adamawa, Benue, Delta, FCT, Imo, Kaduna, Lagos, Oyo, Rivers, and Sokoto States.
Data accessibility	Repository name: Harvard Dataverse Data identification number: https://doi.org/10.7910/DVN/4JIRV9 Direct URL to data: https://dataverse.harvard.edu/dataset.xhtml?persistentId=doi:10.7910/DVN/4JIRV9
Related research article	[1] O. F. Olagunju, D. Kristófersson, T. Kristjánsson, and T. Tómasson, “Technical efficiency of African catfish production in Nigeria: An analysis involving input quality and COVID-19 effects,” *Aquaculture Economics & Management*, pp. 1–27, Jun. 2023, doi: 10.1080/13657305.2023.2222687

## 1 Value of the Data

• The aquaculture sector can benefit from the dataset by gaining insights into the resilience and adaptability of catfish farming during challenging times like the COVID-19 pandemic. It can provide a foundation for theoretical considerations of the mechanisms that contribute to the sector's ability to navigate challenges, serving as a valuable case study for enhancing overall resilience and sustainability in aquaculture.
• Researchers can use this data to conduct in-depth studies on African catfish production trends, efficiency, and challenges. They can analyse the impact of the COVID-19 pandemic on the industry, identify best practices, and develop strategies for sustainable catfish farming.
• Industry stakeholders, including catfish farmers, input suppliers (e.g., feed and equipment), and processors, can gain insights from the data to optimise their operations. It can help them make informed decisions on resource allocation, production techniques, and market positioning.
• Economic theories related to market dynamics and pricing mechanisms can be applied to understand how changes in demand and supply influenced catfish prices before and during the pandemic. This could also include an examination of how market structures may have shifted during this period.
• Policymakers can use these data to formulate evidence-based policies and regulations that support the growth and sustainability of the catfish industry. It can inform decisions related to subsidies, environmental management, and market access.
• Developing countries with growing aquaculture sectors can learn from Nigeria's experience. They can adopt successful practices and avoid common challenges by studying the data. This knowledge exchange can enhance the development of the aquaculture sectors in other countries.

## 2 Data Description

Data from 609 catfish farms were validated and accepted for inclusion in the dataset [2]. The most recent available data were obtained from the farms. All the farms were able to provide their pre-COVID operation data, while only 509 of them reported on operations for the COVID period mostly because they had discontinued operations during that period. The detailed record of removed data could not be adequately tracked as there was active monitoring during the data collection. Farm data with discrepancies, uncertainties, or misreported key production data after clarifications and consultations were removed and data collection continued. In total, about 50 datasets were removed.

### 2.1 The cleaned raw data

Structurally, the questionnaire was classified into one background and four main sections addressing Pre-COVID Production, COVID Production, Management, and Socioeconomic data. The background section elicited information useful for identification and site location. This was followed by the production evaluation data for the two periods as well as the management and socioeconomic data of the farms. In the data spreadsheet (Survey data on catfish farming in Nigeria before and during COVID.xlsx), the data were first arranged based on the different periods with the pre-COVID data arranged in the first 609 rows followed by the COVID period data, making a total of 1118 rows of data. The first series of columns focused on the production data (F to BD) and their derived estimates (Q-T, AZ, BE-BQ). This was followed by the management and the socioeconomic data. While the production data were obtained separately for the two periods, the management and socioeconomic data of the respective individual farms were the same. This should be considered when analysing and interpreting the management and socioeconomic data for the different periods. The production data was collected for a complete production cycle for each period which lasts usually for three to six months, although during COVID, it could extend beyond six months. The production evaluation data includes the stocking and harvest dates; fingerlings quantity, price and source; cost, size and type of pond stocked with the specified fingerlings; details about the feed used including commercial feed and locally compounded feed; maintenance and other costs (Maint-Other cost) which include costs of fertilizers, water supply, electricity, treatment and maintenance of ponds; and lastly, harvest and revenue data.

**Fig. 1:**
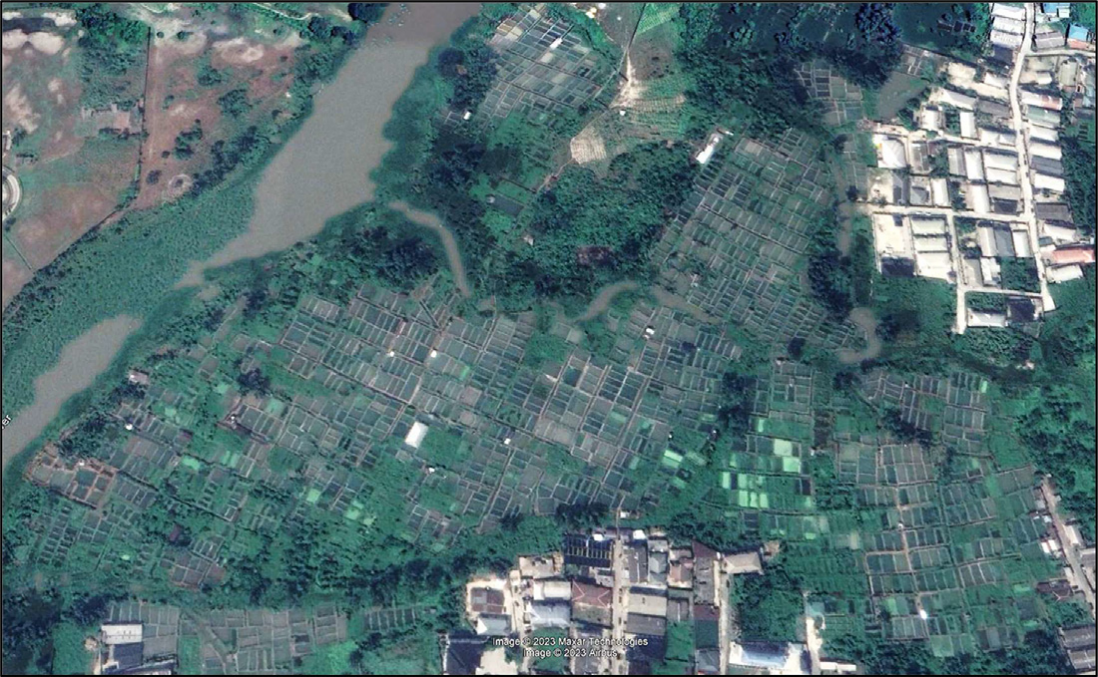
Google Earth imagery of a fish farm cluster in Nigeria covering about 30 hectares with many small pond units (the smaller ones are about 70 m^2^ each). Each farmer may have 1 to 5 ponds in the cluster. Measurements made using Google Earth measuring tool.

Data relating to management (BY-CQ) includes the number and cost of labour engaged and the nature of their engagement (household or hired), farm type, number of cycles covered yearly, total annual production, product forms and market type. The labour characteristics are presented in two ways: (1) based on engagement, including household labour (Household), or (2) if only hired labourers were engaged. An additional column also provides information on whether the household labour was paid or unpaid, with hired labourers classified as paid under this column. The farm types considered are mainly three: backyard (in-house) farms, farm site (outside the residence) farms, and those who are part of a farm cluster (Fig. 1).

The last set of variables was the socioeconomic and other characteristics of the farm (CR-DX). These include data on the sex of the respondent (manager or owner of the farm), types of ponds available on the farm (this includes all pond types on the farm), age range, education level, and major occupation of the farmer, farming experience, source of technical support, constraints, water source, ownership status, types of farm records and production technique based on water usage. Lastly, this section includes information on farmers known to have discontinued fish farming and the causes of their departure.

The question soliciting whether the farmer operated during the COVID period and the impact of COVID on the farmers’ operations (BR-BX) comes immediately after the production data but should be treated like the management and socioeconomic data in that a single response was provided by each farmer for both the pre-COVID and COVID periods.

### 2.2 Calculated variables

The calculated variables include other costs (Maint-Other cost), labour cost, fixed cost and the production scale of the farms (Q-T, AZ, BE-BQ, CG, CJ). Maint-Other cost is the sum of the costs of fertilisers, water supply, electricity, treatment and maintenance of ponds. Nevertheless, each component of other costs can be used independently. The labour cost in the survey was provided as the monthly wage of hired workers. This was prorated based on the annual production of the farm to determine the labour cost for the cycle. In the case of the small-scale farmers who often rely on family labour, the labour cost was taken as an opportunity cost for the farmer as reported by Ali et al. [3]. In such instances, the next best value below the utility derived by other farms was used [4]. Labour measurements in man-days in aquaculture are typically calculated by considering the duration of the culture period and the intensity of the culture system (intensive, semi-intensive or extensive [5]. The length of the production cycle is provided in the dataset and all the catfish farms surveyed practiced intensive farming. Other related data included the number of labourers and the nature of their engagement.

The depreciated cost of the pond or the amount leased for the period was provided as the fixed cost for owned and leased farms, respectively. In cases where the farmers could not provide the cost of construction or purchase of pond, value of the nearest alternative was used based on the pond size and type provided. The annual production for each farm was directly obtained from the data but this was further reviewed in line with the production quantity reported per cycle and the duration of each cycle as reported by the farmer.

### 2.3 Integrated calculation and validation checks

The data was validated through calculations of several variables which were added to the questionnaire (Table 1). In addition to being used as validation checks, they are relevant and useful values retained in the data. The questionnaire template (Nigeria Catfish Fish farm_states - ODK xlsform.xlsx) with the validation checks and the sample survey form (Survey of catfish farms in Nigeria-form.pdf) have been shared online at https://doi.org/10.7910/DVN/4JIRV9, Harvard Dataverse.

**Table 1:** Included validation variables and calculations used in the survey.

Variable	Meaning	Calculation
totseed.cost	Fingerlings cost	Number of fingerlings X unit cost of fingerlings
days.culture	Total days of culture	Stocking date minus harvest date
fishprice	Price of fish per kg	Revenue / harvest quantity
harvestplus	Harvest plus	Reported harvest plus the quantity reserved
revenueplus	New revenue with reserved fish considered	Harvestplus X Price
fcr	Feed Conversion Ratio	Feed quantity / quantity harvested
seed.harvest	Seed to Harvest Ratio	Seed quantity stocked / quantity harvested
feedc.kgfeed	Per kg cost of feed	
feedc.kgharv	Cost of feed used to achieve 1 kg fish	Feed cost / quantity harvested
seedwt	Fingerlings weight (g)	Fingerlings no. X Average unit weight
wtgain	Weight gain	Harvest weight minus Fingerlings weight
fcrplus1	Feed Conversion Ratio – Plus1	Feed Conversion Ratio calculated using Weight gain
fcrplus2 wt HvP*	Feed Conversion Ratio – Plus2	Feed Conversion Ratio calculated using Weight gain based on harvestplus

⁎ Was not included in the initial survey calculation but later calculated in the Excel sheet.

## 3 Experimental Design, Materials and Methods

### 3.1 Sampling design

The sampling design followed the approach of Subasinghe et al. [6], who employed a multi-stage sampling procedure that combined both probability (random) and non-probability (purposive) techniques [7] in sampling fish farms in Nigeria.

Stage I: Purposive selection of state with due consideration given to (i) geographical or regional representation, taking into account their production and agroecological characteristics relative to close non-selected states; (ii) availability of adequate number of catfish farms that allows for a reasonable random selection of a sample size; and (iii) security concerns.

Stage II: Purposive selection of main production areas within each state (Table 2). In collaboration with state supervisors and local experts, major production areas were identified for consideration. This ensured that the survey covers regions that are representative of the state's overall catfish production landscape. In addition, the survey can then concentrate efforts on areas that play a crucial role in the state's aquaculture output.

**Table 2:** Data collection areas under the different states selected for the survey.

Region/Zone	State	Local Government Areas (LGAs)/Area Councils covered
**North**		
North-Central	Benue	*Gboko, Makurdi and Otukpo*
North-Central	FCT	*Abuja Municipal, Bwari, Gwagwalada and Kuje*
North-East	Adamawa	*Demsa, Girei, Numan, Mubi North, Mubi South, Yola North, and Yola South*
North-West	Kaduna	*Chikun, Igabi, Kaduna North, Sabon-gari and Zaria*
North-West	Sokoto	*Dange Shuni, Kware, Sokoto North and Sokoto South*
**South**		
South-East	Imo	*Ideato North, Ikeduru, Mbaitoli, Nwangele, Owerri Municipal, Owerri North and Owerri West*
South-South	Delta	*Ethiope West, Isoko North, Isoko, South, Sapele and Uvie*
South-South	Rivers	*Obio/Akpor, Port Harcourt*
South-West	Lagos	*Agege, Alimosho, Amuwo-Odofin, Apapa, Ajeromi, Badagry, Ifako-Ijaye and Oshodi-Isolo*
South-West	Oyo	*Akinyele, Egbeda, Ido, Ibadan North, Ogbomoso North, Oyo East and Oyo West*

Source: Field survey (2021/2022).

Stage III: Random sampling approach was employed to select individual farmers to participate in the survey from the selected areas. In each state, farmers were randomly drawn from collated lists obtained from government agricultural agencies, feed suppliers, and relevant associations.

To avoid overrepresentation of cluster farms and to ensure a more diverse and representative sample, purposive exclusion principle was adopted by limiting the number of farms that can be sampled in a particular cluster. The number of farms in clusters is usually high, and if not regulated in data collection, the majority of the data collected might be dominated by cluster farms*.* A dedicated survey could be carried out focusing only on cluster farms and their performance.

**Fig. 2:**
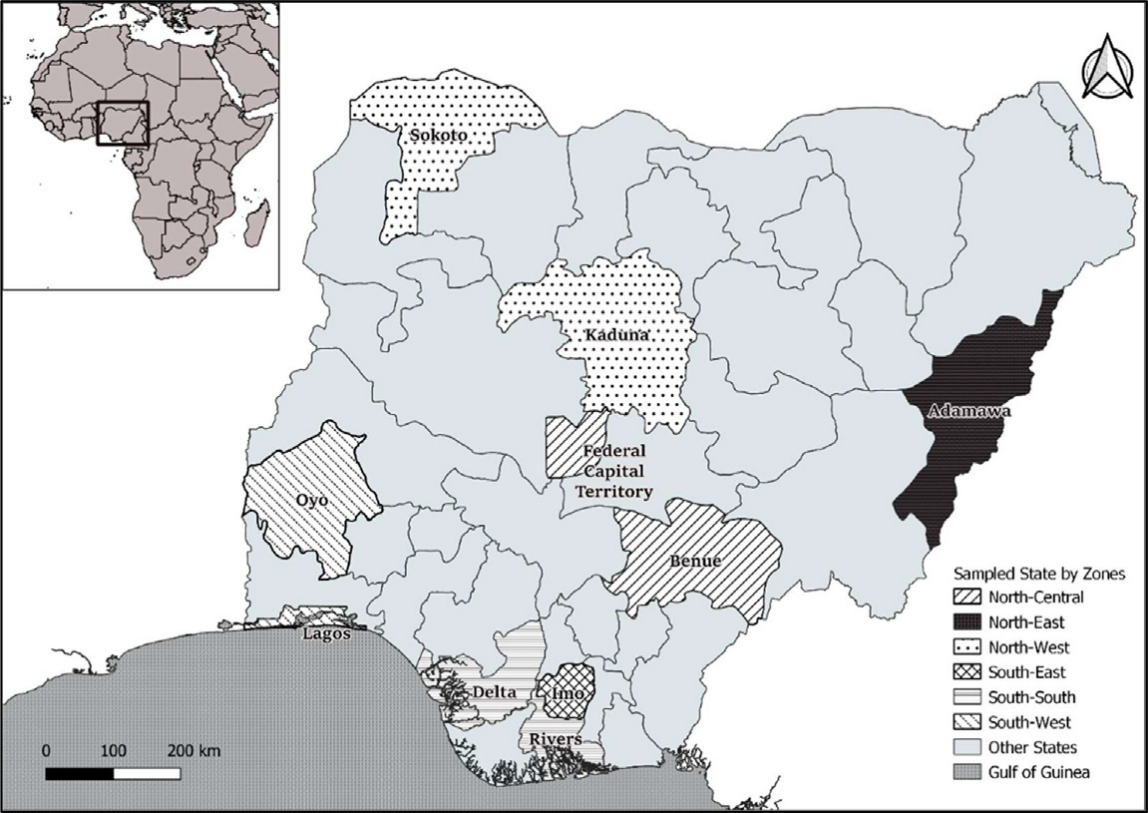
A map of the Federal Republic of Nigeria indicating the zones and states where the survey was conducted. Adapted from Olagunju, et al. [1], with modifications.

A total of 7296 farmers were collated from the farmers lists (Table 3). For such a population size, the minimum sample size should be 365 at 95 % confidence interval and 5 % margin of error. We however increased our target sample size beyond this number to allow for more representativeness of the sample and improve the generalizability of study findings to the broader population. Larger datasets are less sensitive to the influence of outliers or extreme values, ensuring that the results are less likely to be distorted by individual observations. Overall, using the multistage approach, a total of 609 catfish farmers data were sampled covering 10 states from the 6 geo-political zones and 2 regions of the country (Fig. 2).

**Table 3:** Sample size estimation and the number of samples collected from the states surveyed in Nigeria.

Region/Zone	State	Farmers No.	Calculated minimum sample size	Pre-COVID	COVID
**North**		**2693**	**135**	**281**	**235**
North-Central	Benue	400	20	52	34
North-Central	FCT	497	25	62	59
North-East	Adamawa	508	25	64	52
North-West	Kaduna	1110	56	57	44
North-West	Sokoto	178	9	46	46
**South**		**4603**	**230**	**328**	**274**
South-East	Imo	201	10	46	44
South-South	Delta	621	31	72	60
South-South	Rivers	287	14	64	58
South-West	Lagos	3000	150	74	57
South-West	Oyo	494	25	72	55
**Grand Total**		**7296**	**365**	**609**	**509**

Source: Field survey (2021/2022). Adapted from Olagunju, et al. [1], with modifications.

### 3.2 Survey design

The survey was carried out through a series of interviews conducted between May 2021 and February 2022. The pre-COVID data comprises the most recent information obtained from farms prior to 2020, whereas the COVID period data includes information from the years 2020 and 2021. The survey was conducted using the Open Data Kit (ODK) platform. A comprehensive questionnaire was designed to capture a wide range of information related to African catfish farming in Nigeria. The questionnaire employed was an adaptation of the one utilised in the preliminary study conducted in the Federal Capital Territory [8]. It was structurally adjusted to comprehensively capture data for both the pre-COVID and COVID periods. The questionnaire included variables such as input and output quantities, socioeconomic factors, market dynamics, feed types, challenges faced by farmers, production scale, and farmers' experience levels. A team of trained enumerators was engaged to administer the surveys. They were equipped with the necessary skills to effectively conduct interviews with catfish farmers. Meetings and training sessions were conducted with enumerators in various states before the data-collection process to provide them with hands-on training. Additionally, follow-up meetings were held both midway and after data collection to oversee and evaluate the entire process. The data collection primarily took place on-site, with the acquisition of farm photos and geo-locations for the purposes of data verification and cleaning (not included in the dataset for privacy reasons). The enumerators received training on survey protocols and techniques for ensuring data accuracy.

### 3.3 Data quality management

The data collection process was monitored and facilitated by the corresponding author. It included continuous verification and clarification steps. The enumerators worked closely with the participating farmers to ensure that the information provided was accurate and complete. Doubtful or uncertain data were promptly identified and addressed. To enhance data quality, validation checks were integrated into the ODK survey forms. These checks helped in the real-time identification and correction of errors during data entry. The data validation also involved utilising sector-specific expertise and comparing it with related data from individual states. After the data collection, a thorough cleaning process was performed to identify and address any outstanding inconsistencies or inaccuracies. Data that were doubtful due to discrepancies or farmer uncertainty were discarded to maintain data integrity. Furthermore, the approach we adopted in the data collection was aimed at preventing or minimizing cases of missing or invalid data. The use of electronic questionnaire allowed us to make questions compulsory and incorporate calculations that would signal the occurrence of any anomaly. Nevertheless, there were instances of missing data points, such as pond costs (see section “Calculated variables” on how we addressed this). Moreover, some data points were empty, not because they were missing but they were not applicable to the farmer. Example of such is “Other costs” which some small producers did not report any data for, they were left empty. The choice of the methodology to use in addressing such missing data is left to the researcher (potential data user) based on the objective of their study and what they intend to use the data for.

## Limitations

While the survey data and enumerators’ feedback suggest that a significant number of farmers withdrew from farming operations during the COVID-19 period, our data cannot directly provide the percentage of farmers who did or did not produce during the pandemic. Nevertheless, the data can be used to make meaningful comparisons between farm operations, performance, and characteristics before and during COVID. This is because, for 48 farms (DataID: PREC562-609), we could not ascertain whether some of them produced during COVID-19 period as they were only asked to provide data for operations before pandemic lockdown, and two farmers’ COVID period data (PREC513 and 517) were removed for incompleteness. However, a proportionate inference on percentage of producers during the periods can be drawn by excluding these 48 farms from the sample and correcting for the two that have incomplete data. We believe that future researchers could further enhance the dataset, specifically by focusing on farmers operating in clusters.

## Ethics Statement

The authors confirm that we have read and followed the ethical requirements for publication in Data in Brief and confirm that the current work does not involve human subjects, animal experiments, or any data collected from social media platforms. Each participant was notified that their responses would be included in a research project and consented to this by completing the questionnaire. To safeguard the privacy of the participants, all collected data were anonymized. Identifying information, such as names and personal details, were replaced with unique codes to ensure confidentiality.

## CRediT authorship contribution statement

**Olanrewaju Femi Olagunju:** Conceptualization, Methodology, Writing – original draft, Formal analysis, Data curation, Writing – review & editing. **Dadi Kristofersson:** Conceptualization, Methodology, Supervision. **Tumi Tómasson:** Conceptualization, Writing – review & editing, Supervision. **Theódór Kristjánsson:** Writing – review & editing, Supervision.

## Data Availability

Technical and Economic Assessment of Catfish Farming in Nigeria (pre- and post-COVID data) (Original data) (Dataverse).

## References

[1] Olagunju O.F., Kristófersson D., Kristjánsson T., Tómasson T. (2023). Technical efficiency of African catfish production in Nigeria: an analysis involving input quality and COVID-19 effects. Aquacult. Econ. Manag..

[2] Olagunju O.F., Kristófersson D., Tómasson T., Kristjánsson T. (2023). Technical and economic assessment of catfish farming in Nigeria (pre- and post-COVID data). Harv. Dataverse.

[3] Ali H., Rahman M.M., Murshed-e-Jahan K., Dhar G.C. (2018). Production economics of striped catfish (Pangasianodon hypophthalmus, Sauvage, 1878) farming under polyculture system in Bangladesh. Aquaculture.

[4] Greenlaw S.A., Shapiro D. (2018). https://openstax.org/details/books/principles-economics-2e.

[5] Jadhav U. (2009).

[6] Subasinghe R. (2021).

[7] Bluman A.G. (2019).

[8] Olagunju O.F., Kristófersson D., Tómasson T., Kristjánsson T. (2022). Profitability assessment of catfish farming in the Federal Capital Territory of Nigeria. Aquaculture.

